# Pollen Exposed to Aerial Pesticide Spray Is a Major Exposure Pathway for Stingless Bees

**DOI:** 10.1002/ece3.73541

**Published:** 2026-04-19

**Authors:** James P. Hereward, Brian J. Johnson, Rachele Wilson, Tobias Smith, Melissa C. Graham, Darren Alsemgeest, Gregor J. Devine, Michael J. Furlong

**Affiliations:** ^1^ School of the Environment The University of Queensland, UQ St Lucia Queensland Australia; ^2^ Mosquito Control Laboratory QIMR‐Berghofer Medical Research Institute Brisbane Queensland Australia; ^3^ School of Environment and Science Griffith University Brisbane Australia; ^4^ Entomology Laboratory, Public Space Operations Brisbane City Council Brisbane Queensland Australia

**Keywords:** *Bti*, DNA metabarcoding, mangrove, methoprene, mosquito, pesticide, stingless bee

## Abstract

Testing possible exposure routes of pesticides is essential for assessing the risks to pollinators. In Australia, the pesticide *Bacillus thuringiensis* var. *israelensis* (*Bti*) is widely used in mosquito control programs and applied aerially to mangrove and saltmarsh habitats. *Bti* formulations contain both bacterial spores and toxins, allowing detection using DNA‐based methods, whereas bee foraging behaviour can be quantified by pollen DNA metabarcoding. We combined these approaches to investigate both the foraging behaviour of stingless bees and their potential exposure to *Bti* in coastal mosquito habitats. Sentinel hives of the stingless bee *Tetragonula carbonaria* (Meliponini) were placed at two field sites subjected to routine aerial *Bti* treatments. Stingless bee foraging behaviour was assessed by ITS2 metabarcoding of pollen collected from returning foragers, and a *Bti*‐specific qPCR assay was applied to the same pollen samples, hive stores and the bodies of foraging bees after pollen removal. ITS2 metabarcoding revealed that foragers predominantly visited *Avicennia marina* (grey mangrove; Acanthaceae) flowers during the early exposure period, a period of peak flowering in *A. marina* , shifting to *Schinus terebinthifolia* (Brazilian peppertree; Anacardiaceae) and other non‐mangrove species later in the study. *Bti* was detected in pollen loads from 27% of pollen samples (*n* = 184) and 4% of adults (*n* = 287) after pollen removal, identifying contaminated *A. marina* pollen as the primary exposure pathway in the studied environments. A single pollen pot in one of the eight tested was positive, suggesting potential accumulation of *Bti* in hive stores. Detected *Bti* concentrations matched the expected concentrations from field applications and likely pose little risk to stingless bees, but our findings highlight exposure mechanisms that are likely to be significant for pesticides with greater toxicity. The *Bti* qPCR system developed here provides a way to experimentally manipulate and further investigate insecticide exposure pathways for stingless bees.

## Graphical abstract

We conducted a molecular ecology experiment to determine foraging of stingless bees and potential exposure routes for aerially applied pesticides. We found that 27% of forager bees brought back *Bti* to the hive, and that this is a major route for pesticide exposure.

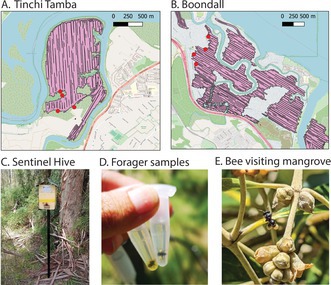

## 1 Introduction

Australian native stingless bees (Meliponini) are culturally, economically and environmentally significant, producing honey and pollinating native and crop plants (Heard and Dollin 2000; Halcroft et al. 2013; Fijn 2014). As managed pollinators they are commonly used alternatives to the European honeybee (*Apis melifera*, Apini) (Halcroft et al. 2013), which is increasingly under threat in Australia following the recent introduction of the Varroa mite (Le Breton et al. 2025). Very little is currently known about the sensitivity of Australian stingless bees to pesticides; however, and this presents challenges to their widespread use in agriculture. A key requirement to understanding the risk of pesticides to stingless bees is to understand exposure routes and exposure risk, but to date most research has focused only on *Apis* bees (Bireley et al. 2019; Thompson and Pamminger 2019; Raine and Rundlöf 2024).

The lack of data on non‐*Apis* species is partly due to the strong focus on European honeybees as pollinators, and to regulatory requirements that often mandate testing of pesticides on *A. mellifera* but not other species (Johnson et al. 2024). There is evidence that stingless bee species may be more susceptible to pesticides than honeybees (Barbosa et al. 2015; Lourencetti et al. 2023; de Castro Lippi et al. 2026). The Meliponini and Apini diverged in the late cretaceous likely at least 80 mya and stingless bees and honeybees evolved advanced social behaviour independently from a primitively social ancestor (Cardinal and Danforth 2011). Stingless bees are therefore evolutionarily distinct from honeybees and data generated from experiments with honeybees should likely be interpreted cautiously with reference to stingless bees (Cham et al. 2019).

It has been demonstrated that foraged pollen is a source of pesticides entering honeybee hives (Villa et al. 2000; Chauzat et al. 2006; de Oliveira et al. 2016; Drummond et al. 2018; Ostiguy et al. 2019; Cappellari et al. 2024). In one study, 79 different pesticides were found in 168 pollen samples (Ostiguy et al. 2019). Because of the various chemical analytical methods used, the number of compounds screened, and the detection limits of the chemical assays, pesticide exposure is likely underestimated in many chemistry‐based studies (Toselli and Sgolastra 2020). In stingless bees, one study found 226 chemical pesticides in stingless bee honey (Krishnappa and Sekarappa 2025), but all were measured as below reportable detection limits for human health concerns.

The increasing popularity of stingless bee keeping across suburban areas of Eastern Australia (Halcroft et al. 2013; Heard 2016) has created overlap between stingless bees and mosquito control programs. This has raised concerns about the potential effects of mosquito control programmes on native stingless bees (Johnson et al. 2024). In Australia most mosquito control relies on the use of biorational larvicides, principally the bacterium *Bacillus thuringiensis* var. *israelensis* (*Bti*) and the insect hormone mimic methoprene (as S‐methoprene) (Russell and Kay 2008; Johnson et al. 2020). As *Bti* is a product based on a bacterium, it is possible to detect the presence and concentration of *Bti* in environmental samples using a qPCR assay (Guidi et al. 2010, 2013). This presents a unique opportunity to study the potential exposure routes of stingless bees to pesticides. The qPCR assay is cheaper and more sensitive than chemical assays, and many chemical insecticides are potentially active and able to kill target insects below the detectable rate (as is the case for Methoprene (Butler et al. 2010)).

*Bti* is a subspecies of *Bacillus thuringiensis* that is particularly pathogenic to the aquatic stages of dipteran insects (Roh et al. 2007). It produces six δ‐endotoxins (Cry4Aa, Cry4Ba, Cry11Aa, Cyt1Aa, Cry10Aa and Cyt2Ba) that form a complex parasporal crystalline body with high and specific toxicity to different mosquito species (Ben‐Dov 2014). Mosquito larvae are killed by the spores of *Bti* and the toxins, which are solubilized in the alkaline pH (9–12) of the mosquito midgut. The acidic stomach of the honeybee (pH 5.6 to 6.8) prevents the solubilization of the toxic crystals and spores, and targeted binding assays have demonstrated that *A. mellifera* lack appropriate epithelial receptors for at least Cry1 and Cry2 toxins (Niu et al. 2017), although this has not been confirmed for the toxins present in *Bti*. These two factors may contribute to the reported tolerance of *Bti* in honeybees, but further testing is required for stingless bees and the risk to stingless bees remains unknown (Johnson et al. 2024).

The aim of this study was to assess whether stingless bees interact with mangrove plant species at coastal mosquito treatment sites and to investigate potential *Bti* exposure routes to Australian native stingless bees. We conducted a field study at two mangrove sites (mostly grey mangrove ( *Avicennia marina* )) where aerial mosquito control treatments are routinely applied. Using ITS2 metabarcoding of individual pollen samples collected from returning foragers, we identified which plants the bees had visited. We then applied a *Bti* specific qPCR assay (Guidi et al. 2010, 2013) to determine whether bees acquired *Bti* from these plants and whether it accumulated in hive stores. These results provide critical insights into exposure pathways and pesticide accumulation in stingless bees.

## 2 Materials and Methods

### 2.1 Field Experiment

**FIGURE 1:**
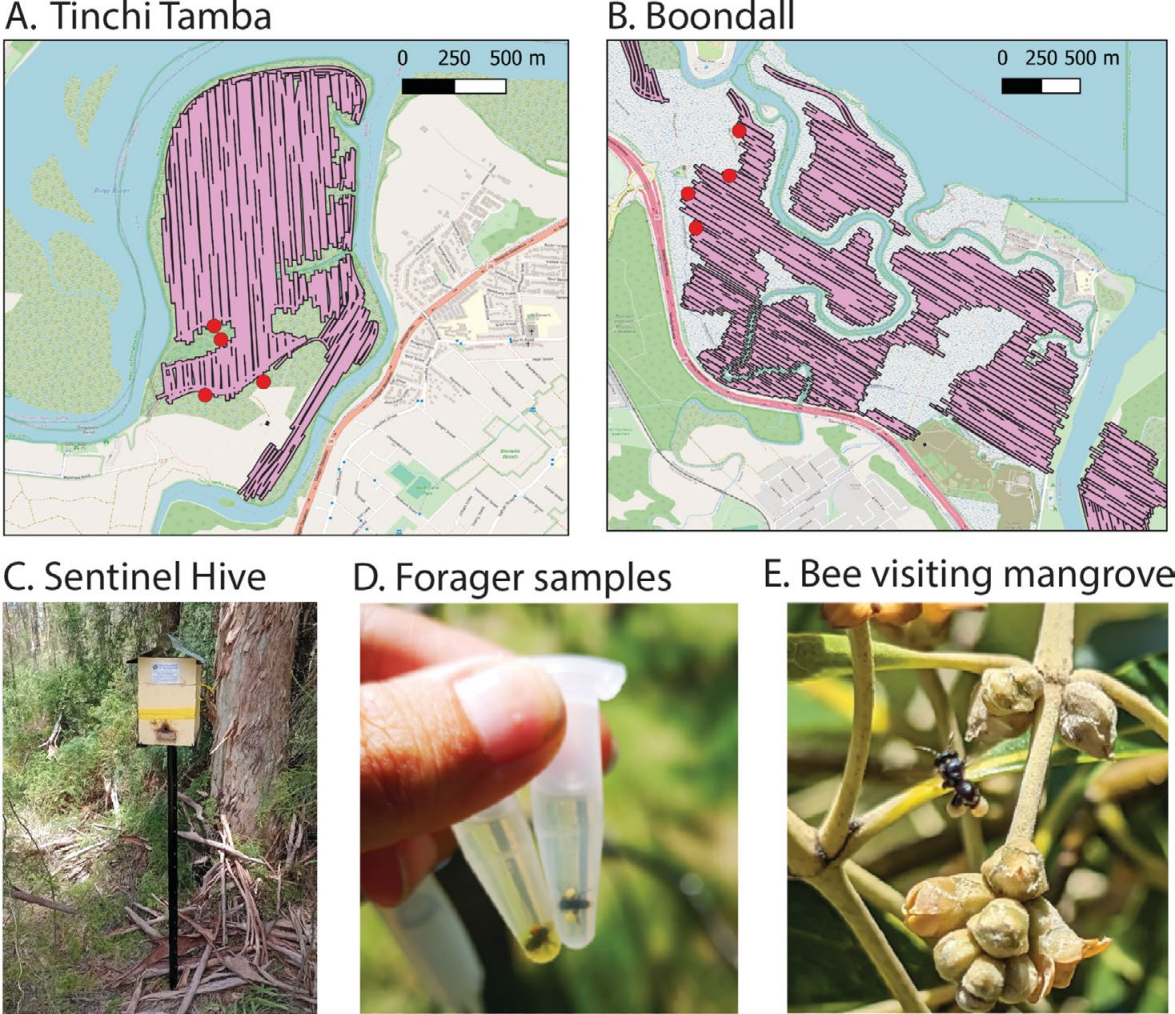
Placement of sentinel hives (red dots) at Tinchi Tamba (A) and Boondall (B), pink lines show the flight path of aerial treatments by helicopter during the study period. Four sentinel hives (C) were placed at each site, and forager bees with pollen loads collected each week (D), bees were observed visiting mangroves during the study period (E).

Eight standard OATH hives of *Tetragonula carbonaria* were placed in the field at two mangrove treatment sites, Tinchi Tamba wetlands (27°17′49.5″ S 153°02′22.5″ E) and Boondall wetlands (27°20′28.2″ S 153°04′53.6″ E), both in the outer northern suburbs of Brisbane, South East Queensland (four hives per site). Hives were placed in the field on 16 February 2023, coinciding with the peak in grey mangrove ( *Avicennia marina* ) flowering, and were removed after 8 weeks when pollen sources became scarce. Prior to this the hives had been maintained in suburban Brisbane sites, all hives were confirmed to have a queen, a good number of workers and healthy brood. Grey mangroves represent the main mangrove species in both treatment areas and are known to be pollinated by *T. carbonaria* . All hives were located within 50 m of the boundary of the nearest aerial treatment site (Figure 1). The timing of hive placement coincided with the period of mosquito treatment activity (Table 1).

**TABLE 1:** Aerial treatment dates and products used.

Site	Date	Time	Active ingredient, formulation (rate)
Tinchi Tamba	17/2/23	07:00	Liquid *Bti*, Vectobac 12 AS (1.2 L ha^−1^)
Tinchi Tamba	22/2/23	06:00	Liquid *Bti*, Vectobac 12 AS (1.2 L ha^−1^)
Boondall	22/2/23	12:00	Liquid *Bti*, Vectobac 12 AS (1.2 L ha^−1^)
Tinchi Tamba	14/3/23	07:00	Liquid *Bti*, Vectobac 12 AS (1.2 L ha^−1^)
Boondall	14/3/23	07:00	Liquid *Bti*, Vectobac 12 AS (1.2 L ha^−1^)
Tinchi Tamba	23/3/23	n.d.	S‐Methoprene, Prolink Liquid Larvicide (0.36 L ha^−1^)

The weight of all hives was recorded upon installation and then weekly for a period of 8 weeks (during the mid‐morning). Returning pollen foragers were sampled from each hive each week to assess foraging activity using metabarcoding and potential contamination by mosquito control products. Foragers with visible pollen loads were taken into individual tubes of ethanol (both the bee and the pollen load) using an aspirator that was cleaned between each sample with 100% ethanol. If less than eight pollen foragers had been collected after 5 min of sampling, then returning foragers with no pollen were sampled. The samples were taken back to the laboratory and stored at −20°C prior to DNA extraction. General hive activity was assessed each week by counting the number of returning foraging bees for a period of 5 min at each hive. At the end of the eight‐week surveillance period, each hive was opened at collection to assess general health (based on bee activity, numbers of workers, presence of a queen and the appearance of the brood) and to obtain brood, honey and pollen samples for DNA analysis to determine if contamination from mosquito control products had occurred and identify botanic sources of hive stores. These samples were transported on ice and stored at −20°C prior to analysis.

### 2.2 Aerial Larvicide Applications

Four aerial larvicide treatments were conducted at the Tinchi Tamba site and three were collected at the Boondall site (Table 1) over the course of the study. All treatments at Boondall, and three of the four at Tinchi Tamba, used liquid *Bti* (*Bti*; Vectobac 12 AS, Valent BioSciences, Libertyville, IL, USA; EPA Reg. No. 73049‐38) as the primary larvicide. This formulation is widely employed in Australian mosquito control programs and consists of an aqueous suspension of *Bti* spores and toxins (strain AM65‐52) with a biopotency of 1200 International Toxin Units (ITU) mg^−1^ (5 × 10^9^ spores mL^−1^). The active component (fermentation solids and solubles) constitutes 11.61% of the commercial product, with the remaining 88.39% comprising non‐active ingredients. Among these, Proxel GXL (Lonza Group Ltd., Basel, Switzerland; dipropylene glycol solution of 1,2‐benzisothiazolin‐3‐one) accounts for 0.10%, while the remainder is withheld as a trade secret. Under operational conditions, the expected field concentration of the product is 1.52 ITU mL^−1^ (applied at 1.2 L ha^−1^ assuming a water depth of 10 cm). Each application covered a consistent area per study site for each application. The total extent of the area treated in each study site is represented by the helicopter flight lines in Figure 1A,B. These lines correspond to the total area of available mosquito breeding habitat within each site.

One treatment at Tinchi Tamba, conducted in the final week of the study, used methoprene (applied as S‐methoprene). Methoprene is an insect growth regulator that interferes with metamorphosis in juvenile insects and some other arthropods, thereby preventing adult emergence (Lawler 2017). Because effective field concentrations are typically very low (0.011–0.020 ppm) (Johnson et al. 2024), environmental detection is difficult, and methoprene exposure was not quantified in this study.

### 2.3 DNA Extraction

For each hives' brood and provisions, DNA was extracted from 3 pupae, 10 μL of larval provisions, 200 μL honey and 200 μL of pollen homogenised in solution with water, respectively. All pupae and larval food stores were removed from propolis casing prior to extraction. These samples were extracted using the Qiagen DNeasy Plant Mini Kit (Qiagen, Germany).

For returning pollen foragers, pollen loads were removed by pulse vortex then centrifuging for 5 min at 20,000 rcf and subsampling 100 μL of dislodged pollen from the ethanol solution into a new tube, the bee was removed and its DNA extracted separately. Ethanol was evaporated from tubes at 37°C overnight and DNA was extracted from the remaining pellets.

Bees were extracted using a DIY silica column method (Ridley et al. 2016) and the pollen was extracted using a combination of CTAB and silica columns (Hereward et al. 2025). For both the bee samples and the pollen samples, 3 mm steel beads and 0.1 mm zirconia beads were used to disrupt the tissue in a TissueLyser II (Qiagen, Germany). For the pollen samples, 300 μL of CTAB buffer was added to each sample with 2 μL of proteinase K and lysed overnight at 55°C following tissue disruption. RNAse was added (2 μL per reaction), and the reaction incubated at 37°C for 30 min. Samples were then cooled and 300 μL of chloroform isoamyl alcohol (25:1) was added, followed by gentle rotation for 5 min. Each sample was separated by centrifugation at high speed (15,000 rcf) for 5 min and the aqueous phase was transferred to a new 1.5 mL Eppendorf tube (Eppendorf, Hamburg, Germany). To the aqueous phase, 300 μL of 4 M GuHCl and 300 μL of 100% ethanol were added, the tubes were gently rotated again for 5 m, and then the entire mix was added to a 96 well silica plate (Epoch Life Sciences, Misouri, Texas, USA), placed on a 2 mL waste collection plate. The Silica plate was centrifuged for 4 min at 4500 rcf to bind the DNA to the silica column. The Silica column was then washed with two washes of 700 μL wash buffer (10 mM Tris–HCl pH 7.5, 80% ethanol), with centrifugation for 4 min at 4500 rcf for the first wash and 8 m at 4500 rcf for the second one. Samples were then eluted with 100 μL of elution buffer (10 mM Tris‐Cl, pH 8.5).

### 2.4 Pollen Metabarcoding

We used the ITS2 region for metabarcoding to identify the plant taxa in pollen samples, and for the brood (old and new), honey, and pollen samples taken from the hives at the end of the 8‐week period. We used ITS‐S2F (Chen et al. 2010) and ITS4R (White et al. 1990) primers, with Illumina (San Diego, USA) adaptor stubs added (FWD TCGTCGGCAGCGTCAGATGTGTATAAGAGACAG, and REV GTCTCGTGGGCTCGGAGATGTGTATAAGAGACAG). We performed duplicate PCR reactions for each sample, and each PCR reaction consisted of 1× NEB Q5 master mix (New England Biolabs), 0.3 μM each primer, and 2 μL DNA. PCR cycling conditions were 95°C for 3 m followed by 35 cycles of 95°C for 40 s, 48°C for 1 min and 72°C for 30 s, with a final extension at 72°C for 5 min (Wilson et al. 2021). The indexing PCR, normalisation and sequencing was performed by the Australian Genome Research Facility (AGRF, Melbourne, Australia) using unique dual indexes and sequencing 384 samples on one 300 bp paired end run on an Illumina Miseq (Illumina, San Diego, USA).

A global database was used (https://doi.org/10.5281/zenodo.3339029) that had been previously created using bcdatabaser (Keller et al. 2020). All raw reads were quality‐filtered (maxEE = 1, no ambitious basepairs) and reads shorter than 100 bp were discarded. Forward and reverse reads were joined, then assigned to amplicon sequence variants (ASVs) using USEARCH 11 including chimera filtering and denoising (Edgar 2010) and classified against the local reference database using global alignments with a threshold of 97% identity. Sample‐specific reads were mapped to ASVs to obtain relative read abundance (RRA) estimates. All ASVs that were not assigned to families of vascular plants were removed from subsequent analyses. Identities were made to the species level, and we manually checked the identifications by searching the ASVs against the NCBI nucleotide database. The metabarcoding data is semi‐quantitative in that the number of sequence reads is proportional to the amount of starting DNA, although there is some bias depending on how well the PCR primers match the sequence of a given species.

### 2.5 *Bti* Detection

The presence of *Bti* was determined by real‐time quantitative PCR (qPCR) analysis of DNA isolated from sampled pollen, forager bodies (adult bees) and sampled hive products. Quantification of *Bti* spores and development of a standard curve from *Bti* stock solution (Vectobac 12 AS, Valent BioSciences, USA) was performed following standard protocols (Guidi et al. 2010, 2013). DNA was extracted as described above for each sample type.

### 2.6 Statistical Analysis

We used generalized linear mixed models (GLMMs) to investigate whether treatment (larvicide application; analysed as days since last treatment), weather variables (weekly mean relative humidity, weekly mean temperature, weekly mean wind speed) and study location (i.e., wetland at which hives were placed) significantly influenced the response variables of hive weight and number of returning foragers. Count data (number of foragers) were modelled using a Poisson error distribution, as these data are discrete, non‐negative integers whose variance is expected to scale with the mean. Overdispersion was addressed by including an observation‐level random effect, a widely used approach that accounts for extra‐Poisson variation by modelling unobserved heterogeneity at the level of individual observations (Harrison 2014). Continuous data (hive weight) were analysed using a Gaussian error distribution. Representative predictors were selected from correlated variables to reduce multicollinearity: temperature, humidity or wind speed for weather, and either week since installation or days since the last treatment for treatment. Hive identity was included as a random effect in all models. The significance of fixed effects was assessed using likelihood ratio tests (LRTs), by comparing nested models with and without the predictor of interest. Test statistics were calculated as the difference in model deviance and evaluated against a *χ* ^2^ distribution with degrees of freedom equal to the difference in the number of parameters. No post hoc tests were performed for models with only continuous predictors, as the effect of each predictor is directly quantified by its estimated coefficient. Daily weather data, summarised at 30‐min intervals, was obtained through the Bureau of Meteorology (www.bom.gov.au) for the closest weather station (Brisbane Airport, Lat: −27.39 Lon: 153.13). All statistical analyses were performed using R (version 4.1.0, R Foundation for Statistical Computing, Vienna, Austria) using the lme4, MASS, MuMIn and multcomp packages.

## 3 Results

### 3.1 Field Experiment

**FIGURE 2:**
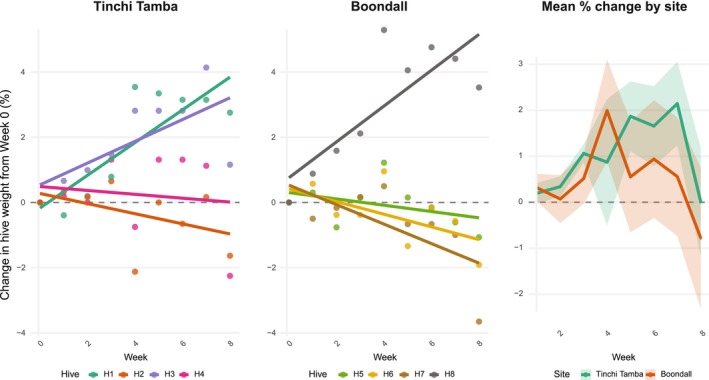
Percent weight change (*y*‐axis) each week (*x*‐axis) at the Tinchi Tamba site (left), Boondall site (centre), and the mean percent change across both sites (right).

Variation in forager activity was explained by variation in mean temperature at the time of each observation (estimate = 0.15, *p* = 0.03) and the week since installation (estimate = −0.19; *p* < 0.001) (Table 2). Forager activity decreased from summer (Week 0) to autumn (Week 8) and was highest during the highest daily temperatures. Time since *Bti* application did not influence forager activity (*p* > 0.05). Colony weights increased slightly with week since installation (estimate = 0.0121; AIC = −123.66, χ ^2^ = 11.313, df = 1, *p* = 0.0007; ∆ *R*_2(marginal)_ = 0.0028; ∆ *R*_2(conditional)_ = 0.985). Median colony weights increased from 5.86 kg (Week 1) to 5.94 kg (Week 7) at Boondall and from 5.69 kg (Week 1) to 5.76 kg (Week 7) at Tinchi Tamba but then decreased in the final week of data collection by 0.02 and 0.14 kg, respectively (Figure 2).

**TABLE 2:** Parameters and statistics for GLMMs.

Model	∆*R* ^2^		LRT			Estimates and post hoc tests	
	Marginal	Conditional	*χ* ^2^	df	*p*	Levels (direction)	*p*
Number of foragers ~ Week + Temperature + (1 | Hive) + (1 | obs_effect)	0.3391	0.9845	32.181	2	***	Week (−0.2372)	***
						Temperature (0.1843)	**0.0066**
Hive weight ~ Week + Site + (1 | Hive)	0.0375	0.9849	20.794	9	**0.013**	Week (0.0034)	?
						Tinchi Tamba < Boondall	?

*Note:* All GLMMs used the random factor of hive and an observation‐level effect where overdispersed. Explanatory power of most parsimonious GLMMs including statistically significant variables is shown as variance of fixed (marginal *R* ^2^) and random effects (conditional *R* ^2^). Significance levels of variables were assessed by likelihood‐ratio tests (LRT) against null models (i.e., random factors only). *p*‐values of < 0.0001 are indicated with ‘***’. ‘?’ represents a failed convergence for one of the nested models in the post hoc comparison. Bold value indicates the significance.

### 3.2 Pollen Metabarcoding

**FIGURE 3:**
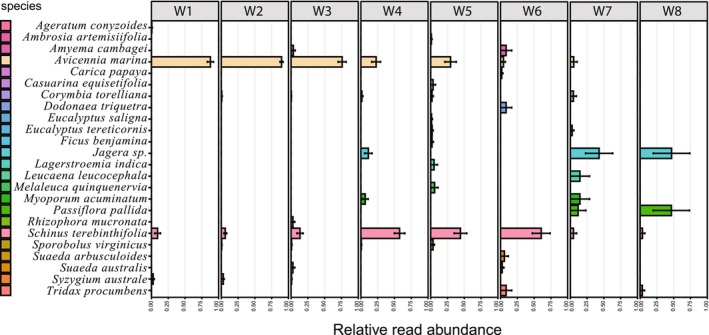
Mean relative read abundance (±SE) of plant taxa identified in bee‐collected pollens by week since hive installation. Bees mostly foraged on grey mangrove ( *Avicennia marina* ) in Weeks 1 to 3 (76%–90%), Brazilian peppertree (Anacardiaceae: *Schinus terebinthifolia*) in Weeks 4 to 6 (44%–61%), *Jagera* sp. (Sapindaceae) in Week 7 (42%) and passionflower (Passifloraceae: *Passiflora pallida* ) in Week 8 (46%).

**FIGURE 4:**
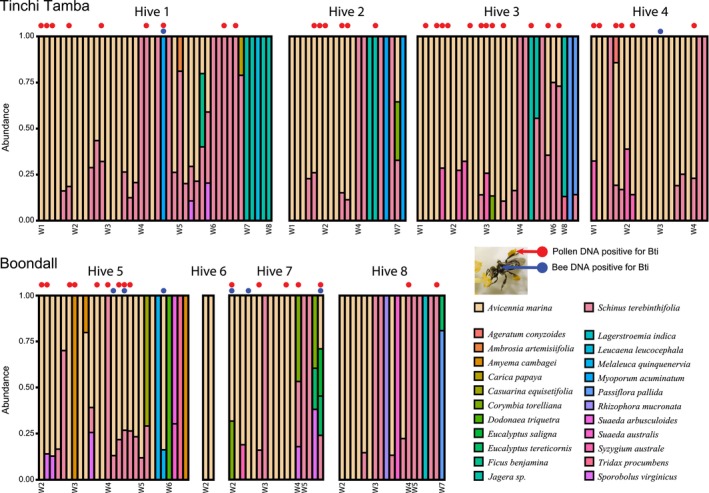
Plant sources identified from forager pollen loads and *Bti* detection in individual foragers (blue dots) and pollen loads (red dots). Each bar is an individual bee returning to the hive with pollen and the colour represents the relative read abundance of each plant species identified expressed as a proportion. For each sentinel hive, Week 1 is on the left and Week 8 on the right.

We recovered a median of 22,302 reads per pollen sample and classified these to 24 plant taxa (Figure 3). Bees mostly foraged on grey mangrove ( *Avicennia marina* ) (76%–90%), the invasive Brazilian peppertree (Anacardiaceae: *Schinus terebinthifolia*) (44%–61%), a *Jagera* sp. (Sapindaceae) (42%), and an introduced passionflower (Passifloraceae: *Passiflora pallida* ) (46%) (Figure 3). Bees collected pollen from grey mangrove ( *A. marina* ) early in the study period but were foraging from other species by week four during the change from summer to autumn (Figure 3). Most bees collected pollen from one or two plant species in a foraging bout, and when pollen from more than one species was collected, one of them was usually more abundant than the other (Figure 4).

### 3.3 *Bti* Detection

*Bti* was detected across both sites and all hives, apart from Hive 6, where pollen forager activity was low and so only two foragers were collected (Table 3, Figure 4). Pollen collected from returning foragers showed the highest frequency of detection, with 26.6% of samples testing positive, compared with only 3.8% in the bees themselves. Mean *Bti* concentrations were similar between adult (1511 spores mL^−1^) and pollen (1262 spores mL^−1^) samples, whereas a single positive pollen store contained a substantially higher concentration of *Bti* (6894 spores mL^−1^). Corresponding mean ITU values were 0.38 ITU mL^−1^ for adults, 0.32 ITU mL^−1^ for pollen and 1.75 ITU mL^−1^ for the hive store. The concentration in the positive pollen store was approximately equivalent to the expected operational field rate of *Bti* at treatment sites (1.52 ITU mL^−1^), whereas concentrations in bees and forager pollen loads averaged about one‐fifth of this rate.

**TABLE 3:** Summary of *Bacillus thuringiensis* var. *israelensis* detections.

Sample type	Number tested	Number *Bti* positive	% Positive	*Bti* conc. (mean; spores mL^−1^)	95% CI (spores mL^−1^)	Mean ITU mL^−1^	95% CI (ITU mL^−1^)
Adults	287	11	3.8	1511	109.5–2913	0.38	0.02–0.73
Pollen	184	49	26.6	1262	274.0–2250	0.32	0.07–0.57
Stores	30	1	3.3	6894	—	1.75	—

*Note:* 1 ITU = LC_50_ concentration for *Aedes aegypti* relative to reference standard. The only reference standard currently available is Valent BioSciences Corp. strain AM65‐52, lot #82‐691‐W5, which has a biopotency of 7992 ITU/mg.

Abbreviation: ITU, International Toxic Units.

*Bti* detections were distributed across pollen samples dominated by a range of plant taxa (Figure 4). The majority (*n* = 39, 73.5%) coincided with pollen loads identified as containing *Avicennia marina* , which was the most common foraging resource across both study sites. In all but three of the positive samples containing *A. marina* , this species was the most abundant pollen source. Positive detections were also observed in pollen loads comprised mostly of *Schinus terebinthifolia*, *Casuarina equisetifolia* (Casuarinaceae), *Melaleuca quinquenervia* (Myrtaceae) and several minor contributors, suggesting that *Bti* contamination was not restricted to a single floral resource. After *A. marina* , *S. terebinthifolia* was most frequently associated with positive detections, occurring in 30.6% (*n* = 15) of *Bti*‐positive pollen samples.

Adult bee samples that tested positive for *Bti* showed a similar association with *A. marina* . Of the eight positive bees with paired pollen samples, six were associated with pollen loads dominated by *A. marina* , one with *A. marina* as a secondary pollen resource and one with a pollen load dominated by *M. quinquenervia* . In half these cases, positives coincided with pollen samples that were also positive for *Bti*, whereas in the other half, bees tested positive despite collecting pollen that was negative for *Bti*. This pattern suggests that while contaminated pollen is the primary route of *Bti* exposure, surface contamination of foragers may also occur independently of pollen carriage.

## 4 Discussion

We found that stingless bees mostly foraged on mangrove flowers during peak bloom, and when aerial mosquito treatments overlapped with this period, pollen was frequently contaminated with *Bti*. At treated sites, about a third of foragers (27%) returned with *Bti*‐contaminated pollen and a smaller percent (4%) were *Bti* positive in the absence of pollen. Although no adverse effects were observed in sentinel hives, and *Bti* is not expected to be toxic to stingless bees (Johnson et al. 2024), our results demonstrate that pollen exposed to aerial pesticide sprays represents a major exposure pathway for stingless bees. This pathway is likely to be the same for other, more toxic, chemicals.

*Tetragonula carbonaria* is known as a generalist (polylectic) pollinator, and it has been recorded foraging from over 300 plant species in orchards (Wilson et al. 2021). In this study we found 24 species of pollen in returning foragers, with a good representation of the plants observed at each site in the species detected. We detected some plants not immediately obvious at the field sites; for example, *Amyema cambagei* (Loranthaceae), a mistletoe of *Casuarina* spp., highlighting the power of metabarcoding bee forager pollen to detect cryptic flora. The pattern of foraging was similar at both sites, with mostly pollen from grey mangrove ( *A. marina* ) being collected initially and decreasing as the study progressed (Figures 3 and 4). This is due to the availability of grey mangrove flowers reducing during the study period as it coincided with the end of the flowering season. As the study progressed and the availability of mangrove pollen reduced, fewer returning foragers were observed carrying pollen, suggesting that without the mangrove flowering there are few abundant pollen sources at these sites. *Tetragonula carbonaria* generally flies at temperatures over 18°C (Heard and Hendrikz 1993), but we found reduced numbers of total foragers as temperatures reduced towards the end of the study period, even when average temperatures were still around 25°C. We therefore confirmed that when placed in a mangrove site, *T. carbonaria* will collect mangrove pollen; however, these sites may not be the most suitable for natural hives due to the seasonal availability of abundant pollen. This is supported by surveys in southeast Australia that have highlighted the absence of *T. carbonaria* from temperate mangrove forests at the southern end of their distribution (Hermansen et al. 2014; Williams 2020).

When the availability of grey mangrove pollen reduced, many foragers collected pollen from *Schinus terebinthifolia*, commonly known as Brazilian peppertree, an environmental weed in SE Queensland. The bees also visited *Leucaena leucocephala* (Fabaceae), *Ambrosia artemisifolia* (Asteraceae), *Passiflora suberosa* and *Ageratum conyzoides* (Asteraceae), all of which are invasive and classified as environmental weeds in the region (https://weeds.brisbane.qld.gov.au/). This highlights how, as generalist foragers, native stingless bees such as *T. carbonaria* will visit invasive species as well as native ones and can therefore potentially play a role in aiding the invasion process (Stout and Tiedeken 2017). The impacts of foraging on exotic species to the pollinator are not well known and likely both species specific and context dependent (Drossart et al. 2017). Because invasive weeds often exploit disturbed edge habitats, they may be disproportionately contaminated with pesticides in the studied environments, particularly along the transition from saltmarsh to coastal forest. When these plants represent attractive foraging resources, they may increase pesticide exposure in stingless bees and other bee species due to the inadvertent contamination of floral resources. This likely explains the general association between *S. terebinthifolia* and positive *Bti* samples in the present study. *Schinus terebinthifolia* rapidly colonizes disturbed bushland, making it abundant along forest edges (Nickerson and Flory 2015), and its flowers are highly attractive to pollinators both within and beyond its native range (Fragoso and Varanda 2011; Colteaux et al. 2013).

We did not experience any hive loss during the study period, and the hive weights—a proxy for colony strength—did not change drastically, with some increasing and some decreasing a small amount throughout the study (Figure 2). This is in line with the expected relative safety of *Bti* for stingless bees (Johnson et al. 2024) due to their acidic gut pH and likely lack of receptors as inferred from honeybees. Methoprene is also unlikely to be lethal to adult stingless bees based on research on other bee species (Deng and Waddington 1997; Tasei 2001) but may impart behavioural shifts in critical activities (Robinson 1985; Robinson and Ratnieks 1987). Better understanding the toxicity of both insecticides therefore requires specific bioassay development for *Tetragonula* and, in the case of methoprene, assays on bee larvae are critical to better understand the safety of these treatments (Johnson et al. 2024).

Because *Bti* is bacterial we were able to quantify it in the pollen loads of returning foragers and found a markedly high proportion (27%) of pollen samples positive for *Bti*, indicating that pollen foraging is a major exposure route by which pesticides may enter stingless bee hives. Although the observed rates of contamination are unlikely to impact native stingless bees due to the presence of physiological barriers well studied in the honey bee ( *Apis mellifera* ), the results highlight a contamination pathway relevant to more toxic pesticides applied in agricultural and other pollinator‐heavy systems. Of these, there is considerable concern regarding systemic pesticides being brought back in foraged pollen by other bee species (Rortais et al. 2005; Raine 2018; Phan et al. 2024), and our results suggest that pesticides applied to flowering plants by spray are likely to be collected by forager stingless bees and brought back. Although only a single sample of pollen stored inside a hive tested positive for *Bti*, the concentration was around five times higher than the mean found in the pollen loads carried by foraging bees, suggesting a potential accumulation of pesticides in hive stores following contamination of floral resources. We found no evidence of *Bti* in the other seven hives, but we only sampled one pollen pot per hive out of many pollen pots, and it is possible that these pots may have been filled prior to the start of the field experiment. Stingless bees feed their brood with a mix of pollen and honey, so our finding of within‐hive pollen stores positive for *Bti* suggests that chemicals brought back by forager bees are likely to be fed to bee larvae.

Our combination of molecular approaches (plant metabarcoding and *Bti* qPCR) and field experimentation has enabled us to gain new insights into pesticide exposure pathways in Australian native stingless bees. We confirmed that stingless bees will visit mangrove flowers during mosquito treatment events, and that they bring *Bti* back to the hive at a high frequency (27%). We suggest that these findings are broadly applicable to pesticides sprayed onto flowering plants. We also suggest that the *Bti* qPCR system outlined here provides a way to experimentally manipulate and further investigate insecticide exposure pathways for pollinators.

## Author Contributions

**James P. Hereward:** conceptualization (equal), formal analysis (equal), funding acquisition (equal), investigation (equal), methodology (equal), project administration (equal), writing – original draft (equal), writing – review and editing (equal). **Brian J. Johnson:** conceptualization (equal), formal analysis (equal), funding acquisition (equal), investigation (equal), methodology (equal), writing – review and editing (equal). **Rachele Wilson:** formal analysis (equal), investigation (equal), methodology (equal), visualization (equal), writing – review and editing (equal). **Tobias Smith:** conceptualization (equal), investigation (equal), methodology (equal). **Melissa C. Graham:** investigation (equal). **Darren Alsemgeest:** investigation (equal), methodology (equal), resources (equal). **Gregor J. Devine:** conceptualization (equal), funding acquisition (equal), writing – review and editing (equal). **Michael J. Furlong:** conceptualization (equal), funding acquisition (equal), supervision (equal), writing – review and editing (equal).

## Funding

This work was supported by the Mosquito and Arbovirus Research Committee.

## Conflicts of Interest

The authors declare no conflicts of interest.

## Data Availability

The DNA metabarcoding data and R script have been uploaded to UQ espace, doi: https://doi.org/10.48610/cf3a023.
